# Thermal Performance Study of Composite Phase Change Material with Polyacrylicand Conformal Coating

**DOI:** 10.3390/ma10080873

**Published:** 2017-07-28

**Authors:** Shin Yiing Kee, Yamuna Munusamy, Kok Seng Ong, Hendrik Simon Cornelis Metselaar, Swee Yong Chee, Koon Chun Lai

**Affiliations:** 1 Faculty of Engineering and Green Technology, Universiti Tunku Abdul Rahman, Kampar 31900, Malaysia; nicolekee88@hotmail.com (S.Y.K.); skong@utar.edu.my (K.S.O.); laikc@utar.edu.my (K.C.L.); 2 Department of Mechanical Engineering and Advanced Material Research Center, University of Malaya, Kuala Lumpur 50603, Malaysia; h.metselaar@um.edu.my; 3 Faculty of Science, Universiti Tunku Abdul Rahman, Kampar 31900, Malaysia; csy@utar.edu.my

**Keywords:** phase change material, PMMA, myristic acid, polyacrylic coating, conformal coating

## Abstract

The composite PCM was prepared by blending polymethyl methacrylate (PMMA) and myristic acid (MA) in different weight percentages. The MA and PMMA were selected as PCM and supporting material, respectively. As liquid MA may leak out during the phase transition, this study proposes the use of two coatings, namely a polyacrylic coating and a conformal coating to overcome the leakage problem. Both coatings were studied in terms of the leakage test, chemical compatibility, thermal stability, morphology, and reliability. No leakage was found in the PCMs with coatings compared to those without under the same proportions of MA/PMMA, thus justifying the use of coatings in the present study. The chemically compatibility was confirmed by FTIR spectra: the functional groups of PCMs were in accordance with those of coatings. DSC showed that the coatings did not significantly change the melting and freezing temperatures, however, they improved the thermal stability of composite PCMs as seen in TGA analysis. Furthermore, the composite PCMs demonstrated good thermal reliability after 1000 times thermal cycling. The latent heat of melting reduced by only 0.16% and 1.02% for the PCMs coated with conformal coating and polyacrylic coating, respectively. Therefore, the proposed coatings can be considered in preparing fatty acid/PMMA blends attributed to the good stability, compatibility and leakage prevention.

## 1. Introduction

The greenhouse effect is the main driving force for switching from fossil fuel energy to various renewable energy sources such as solar, wind, and hydro energy. Solar energy is a popular renewable resources for electricity generation and thermal energy storage (TES). A comparative study on world energy consumption released by the International Energy Agency pointed out that solar array installations will supply about 45% of worldwide energy demand in 2050 [1]. TES is rapidly growing in mitigating energy crises and reducing environmental pollution [2]. In particular, the use of phase change material (PCM) for TES is one of the most prospective techniques [3,4,5,6]. PCM has high latent heat and capable of storing heat energy with a small temperature change during phase transition [7,8]. It stores five to 14 times more heat per unit volume than sensible storage materials such as water, masonry, and rock [9]. Moreover, a significant reduction in storage volume can be achieved using PCM compared to sensible heat storage [10].

PCMs are commonly classified into organic, inorganic, and eutectic [11,12,13,14]. Among these, organic PCMs using fatty acids—e.g., stearic acid (SA) and myristic acid (MA)—are promising. This is due to their characteristics of high latent heat, good chemical stability, and wide melting range [15,16,17]. The main problem with fatty acid is the corrosion of their containers [18]. Several studies have been carried out to prepare form-stable, composite PCMs by encapsulating fatty acid in supporting material. Song et al. [19] developed the form-stable PCMs by incorporating stearic-capric acid into activated-attapulgite (a-ATP). The thermal and chemical reliability was confirmed by analyses of differential scanning calorimetry (DSC) and Fourier transformation infrared (FTIR). Alkan and Sari [20] prepared a series of fatty acid in poly(methyl methacrylate) (PMMA) blends. The form stable PCMs were identified when the blends passed the leakage test whereby no leakage of melted PCM could be found. Chen et al. [21] prepared form-stable PCMs using electrospinning. Field emission scanning electron microscope (FE-SEM) and DSC were employed to study the morphological and thermal properties, whereas the thermal stability and reliability were evaluated through 100 heating-cooling thermal cycles. Thermal stability of PCMs was analyzed by a thermogravimetric analyzer (TGA), as stated by Gasia et al. [22]. They also examined the thermo-physical properties (i.e., latent heat and melting temperature along a number of freezing-melting cycles). Throughout the reported findings, the form stable composite PCMs can be viewed as a candidate to control the corrosion problem and volume change of fatty acid during phase change process [23,24]. 

A wide range of techniques has been developed in dealing with leakage prevention. Li et al. [25] used three types of nano silica powder: hydrophilic fumed silica, hydrophobic fumed silica and precipitated silica to prepare form-stable PCMs. PCMs leakage was blocked when hydrophobic fumed silica and precipitate silica were in a 2.3% and 9.0% mass fraction, respectively. In addition, Ramakrishnan et al. [26] coated the expanded perlite (EP) with hydrophobic silicone, whereas Kheradmand et al. [27] prepared composite PCMs surface coated with four types of waterproofing solution. Other methods focused on the encapsulation in organic particles, such as coating with mixture of colloidal silica and organic acrylate to reduce leakage [28]. 

In the present study, the composite PCM will be prepared by encapsulating the myristic acid (MA) into poly(methyl methacrylate) (PMMA), which was used as supporting material. PMMA is one of the polymers that are commonly used owing to its compatibility with fatty acid and good thermal stability [29]. However, the major drawback is the leaking issue. In line with this, two types of coating—polyacrylic coating and conformal coating—were proposed to coat the composite PCM for leakage prevention. The two coatings have high transparency and UV resistance, are stable to environment condition, and exhibit good barrier properties to moisture [30]. Despite several studies on polyacrylic coating and conformal coating, there is a lack of research on the application of these coatings for composite PCM. The goal of this paper is to prepare and characterize coatings on form-stable PCM (MA/PMMA) for solving the leakage problem. The performance of PCM with coating will be compared to that without coating. As a first step, the leakage resistance will be investigated through a leakage test technique. Then the chemical compatibility and thermal properties will be characterized using DSC, FTIR, and scanning electron microscope (SEM). Thermal stability will be measured via TGA method, whereas the reliability will be evaluated by 1000 melting and freezing cycles.

## 2. Experimental

### 2.1. Materials

Myristic acid (MA) and chloroform were purchase from R&M Chemicals (Semenyih, Malaysia). Thermal properties of MA (i.e., melting point, freezing point, latent heat of melting, and latent heat of freezing) are given as 55.97 °C, 53.02 °C, 213.43 J/g, and 214.67 J/g, respectively. The supporting material Polymethyl methacrylate (PMMA) was purchased from Sigma Aldrich, Subang Jaya, Malaysia. 

### 2.2. Preparation of the Composite PCMs 

The composite PCM (MA/PMMA) was prepared by solution blending method. MA and PMMA were dissolved in chloroform in different beakers at room temperature. The concentrations of PMMA and MA in chloroform are 0.01 g/mL and 0.05 g/mL, respectively. The MA solution was added to PMMA solution drop-wise. The mixture was dried naturally for 48 h followed by drying in a universal oven (UN110, Memmert GmbH + Co. KG, Schwabach, Germany) at 40°C for 48 h. Then, the composite PCM blends were prepared at different weight percentage of MA (20%, 40%, 60%, and 80% *w*/*w*). All blends were immersed into the coating solution for 10 min and subject to sonication in an ultrasonic water bath (Elmasonic S180(H), Elma Schmidbauer GmbH, Singen, Germany) for five minutes to eliminate bubbles. The coatings used in the present study were silicone conformal coating (Dow Corning 1-2620) and polyacrylic coating. The former was purchased from Celtite Sdn Bhd, Johor Bahru, Malaysia, whereas the latter was synthesized via emulsion polymerization with methyl methacrylate, butyl acrylate, and acrylic acid as monomers. The polymerization was performed in a five-necked reactor flask with the stirrer speed of 600 rpm under semi-batch process at 75 °C.

### 2.3. Characterization

**Figure 1:**
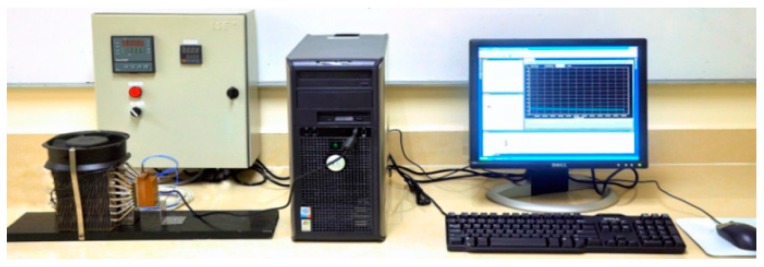
In-house thermal cycling test system.

The leakage test method was adopted from the diffusion-oozing circle test proposed by Ma et al. [31]. The composite PCM samples were uniformly distributed within a 13-mm diameter circle drawn on the litmus papers and put in the universal oven at 65 °C. After two hours of heating process, the leakage area of each composite PCM blends on the litmus paper was measured. The leakage percentage (η) was calculated by

(1) $$\eta =A_{L}/A_{L}\times 100\%,$$

where *A*_L_ and *A*_R_ denote the leak area and reference area, respectively. The value of η may exceed 100% when the coating has a larger pole diameter that allow exudation [31]. Infrared spectra of the samples were acquired from a FTIR photospectrometer (Nicolet 8700, Thermo Fisher Scientific Inc., Waltham, MA, USA) using KBr disk. This is to obtain the information of functional groups in the samples and to study the interaction of MA and PMMA. Another photospectrometer with attenuated total reflectance (Nicolet iS10 FTIR-ATR, Thermo Fisher Scientific Inc., Waltham, MA, USA) was used to record the spectra of samples with coating. Attenuated total reflectance (ATR) can analyze the composite PCM with coating in their natural states without destroying the coating. All the analyses were carried out in a wavelength range of 4000 cm^−1^ to 401 cm^−1^ with scanning resolution of 2 cm^−1^. The thermal properties of samples such as melting point and latent heat were measured by a differential scanning calorimetry (DSC 1, Mettler Toledo Inc., Greifensee, Switzerland). The analysis was performed at a temperature range of 25–100 °C and 5 °C/min heating rate under a constant stream of nitrogen gas at the flow rate of 20 mL/min. In order to determine the thermal decomposition temperatures of samples, a thermogravimetric analyzer (TGA SDTA851E, Mettler Toledo Inc., Greifensee, Switzerland) was employed under dynamic nitrogen flow at a heating rate of 10 °C/min in the range of 25–700 °C. The morphologies of samples at different magnifications were captured by a scanning electron microscope (JSM 6701F, JEOL Inc., Peabody, MA, USA). Each sample was deposited on a disc with scope tape and examined after sputter coating with thin layer of platinum so as to avoid electrostatic charging and poor image resolution. Furthermore, thermal reliability of the samples was evaluated through a 1000 times repeated thermal cycling using an in-house thermal cycling system [32], as shown in Figure 1.

## 3. Results and Discussion

### 3.1. Leakage Analysis

Four samples prepared at different proportions of MA and PMMA were evaluated. The corresponding results are presented in Table 1. The leakage percentage increased with increasing weight percentage of the MA for all samples. The weight percentage of PMMA that provides physical hooks between the chains and form a network structure to block the fatty acid molecules was reduced. As a result, a continuous and dense network cannot be formed, thus leakage was seen.

The effect of the coatings in leakage prevention is apparent in Table 1. For instance, the leakage percentage of sample 4 was reduced by at least 80% after coating. This is attributed to the coating films that form a barrier to encapsulate the fatty acid and prevent leakage. It is also noticeable that the polyacrylic coating performs slightly better than the conformal coating in term of blockage. No leakage was seen when the polyacrylic coating was used in sample 3, as indicated by the η value of 100%, compared to that of conformal coating. Nevertheless, the use of conformal coating in sample 3 is considered as acceptable as the leakage did not exceed the maximum η value of about 130% [33]. Along the same line, only polyacrylic coating can be chosen for sample 4 for the preparation of form-stable PCMs. The samples that can be used as form-stable PCM (FSPCM) were marked bold in Table 1. 

**Figure 2:**
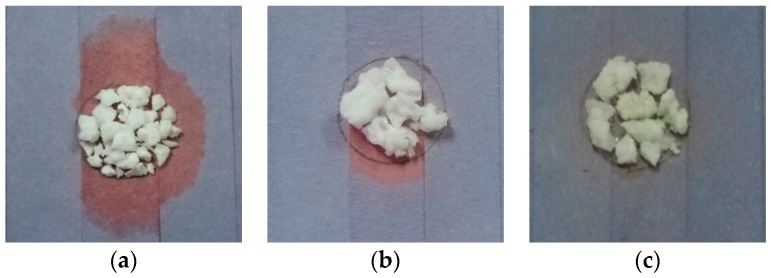

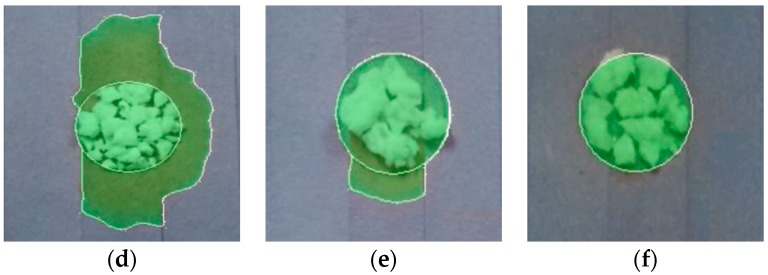
Leakage comparison for sample 3 and calculation of η after the heating: (**a**,**d**) without coating; (**b**,**e**) with conformal coating and (**c**,**f**) with polyacrylic coating.

Each sample was placed on a blue litmus paper and heated at 65 °C for 2 h. Leakage can be clearly seen in Figure 2a for sample without coating as the litmus paper has turned into red. On the other hand, sample with conformal coating remains mostly solid as shown in Figure 2b. However, a certain amount of leakage can still be noticed. The leakage was totally avoided, as depicted in Figure 2c, when the polyacrylic coating was applied. 

### 3.2. FTIR Analysis

**Figure 3:**
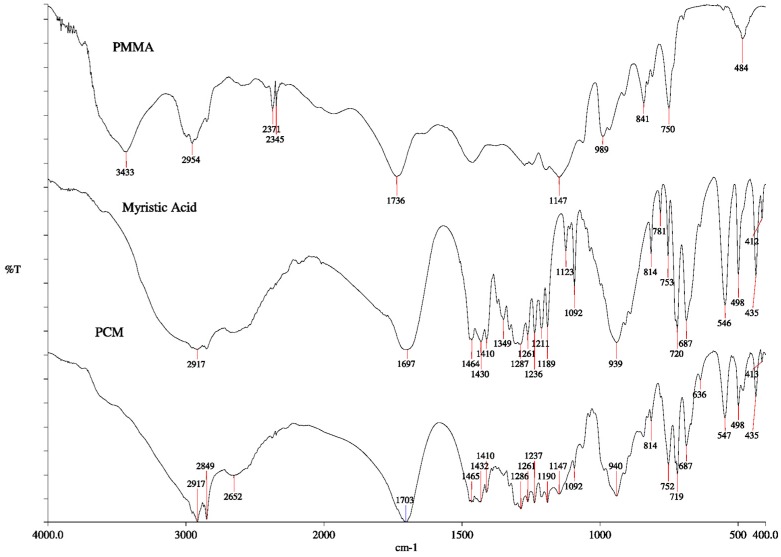
Fourier transformation infrared (FTIR) spectra for the pure polymethyl methacrylate (PMMA), pure myristic acid (MA), and composite phase change material (PCM) (MA/PMMA, 60/40 wt %).

Theprobable interactions between the PCM and coatings were investigated using FTIR spectroscopy. Figure 3 presents the FTIR spectra for the pure PMMA, pure MA and the composite PCM (sample 3 was given as an example). For PMMA, the peaks at 2999–2953 cm^−1^, 1735 cm^−1^, 1458 cm^−^^1^ and 1147 cm^−1^ were assigned to CH stretching, C=O stretching, CH_3_ stretching and –O–CH_3_ stretching vibrations, respectively. For MA, C=O peak occurred at 1697 cm^−1^ and C–H stretching peak occurred at 2917 cm^−1^. The peaks corresponding to the out-of-plane bending vibration and in-plane swinging vibration of –OH functional group of the MA were found at 939 and 720 cm^−1^, respectively. The peaks of PCM are almost similar to MA due to the high mass fraction of fatty acid (60% *w*/*w*). The hydroxyl peak (O–H) of MA in the range of 2500–3300 cm^−1^ has small change due to the interaction between oxygen atom of carbonyl group (C=O) PMMA and hydrogen atom of hydroxyl group of MA. The spectral results in this study were in good agreement with the reported findings in [18,20]. The hydrogen bonding between MA and PMMA increased the compatibility between both components of the composites. However, no new functional group was found.

**Figure 4:**
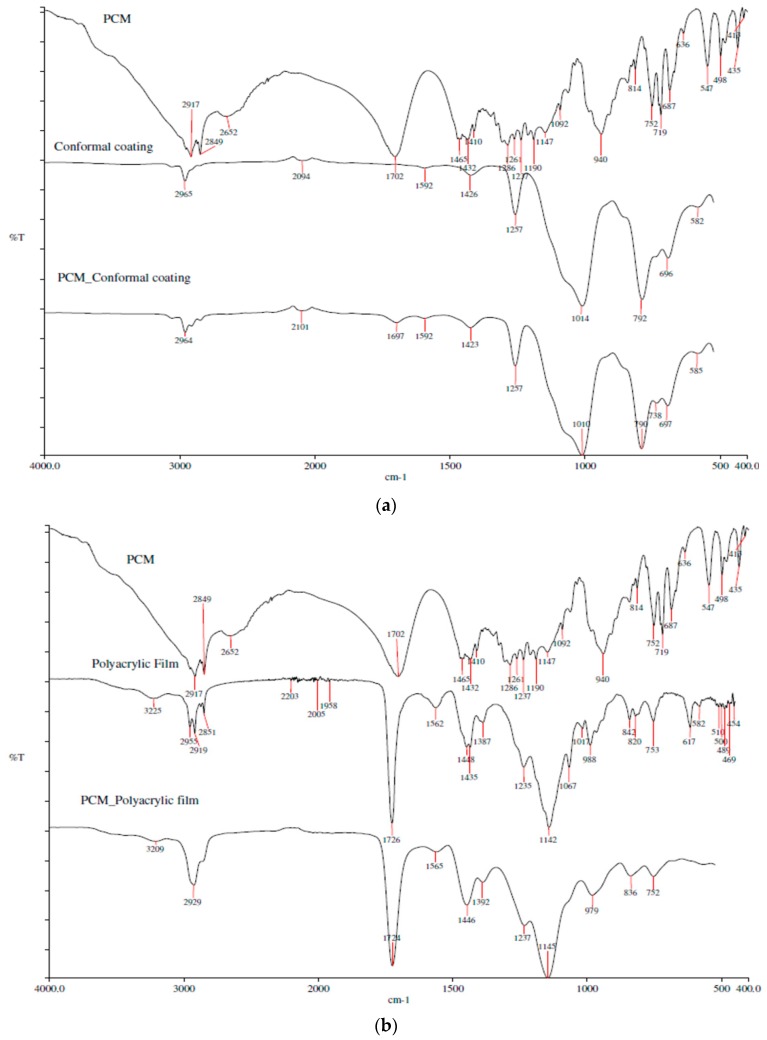
FTIR spectra: (**a**) PCM with conformal coating and (**b**) PCM with polyacrylic coating.

The FTIR spectra for the PCM with coatings are displayed in Figure 4. Figure 4a shows the silicone conformal coating peaks at 2965 cm^−1^, 1426 cm^−1^, 1257 cm^−1^, 1014 cm^−1^, and 792 cm^−1^ were assigned to C–H stretching, C–H bend, Si–CH_3_, Si–O bond, and Si–C, respectively; whereas polyacrylic coating peaks at 3225 cm^−1^, 2955–2851 cm^−1^, 1726 cm^−1^, 1448 cm^−1^, and 1142 cm^−1^ were assigned to O–H stretching, C–H stretching, C=O stretching, CH_3_ stretching, and –O–CH_3_ stretching vibrations, respectively, in Figure 4b. The spectra findings confirmed the good chemical compatibility between the coatings and PCM since only the coating functional groups were detected in the PCM with coatings. For instance, C–H stretching peak for PCM and conformal coating was at 2917 cm^−1^ and 2965 cm^−1^, respectively, and was at 2964 cm^−1^ for the coated PCM. Likewise for polyacrylic coated PCM, C=O stretching peak was at 1724 cm^−1^ compared to the 1702 cm^−1^ for PCM and 1726 cm^−1^ for polyacrylic coating.

### 3.3. Thermal Properties and Thermal Stability Analyses

**Figure 5:**
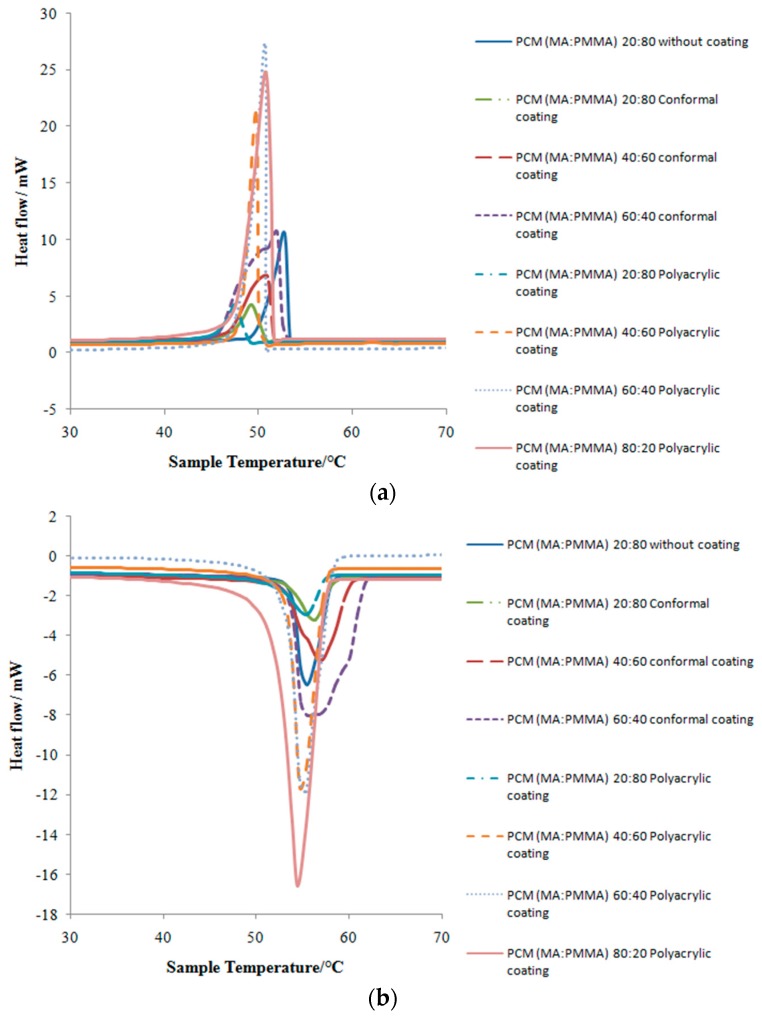
Differential scanning calorimetry (DSC) curves of various samples: (**a**) cooling curves and (**b**) heating curves.

Thermal properties of the FSPCM samples were studied via differential scanning calorimetry (DSC) analysis. The melting point, freezing point, and latent heat storage capacities are shown in Table 2 and Figure 5. The latent heat increased with the weight percentage of MA in all samples (without coating and with coatings). The differences of melting and freezing temperature between composite PCMs and MA were small due to the negligible effect of blending PMMA.

The latent heat of melting and freezing of FSPCMs were reduced after the coating. This is due to the fact that all the MA substance mass in the sample without coating is fatty acid. However, the substance in the coated samples is a mixture of the coating and fatty acid. Consequently, the latent heat determined from the DSC analysis is lower than the theoretical value. It is noteworthy that both coatings did not significantly affect the melting and freezing points. Furthermore, the high latent heat storage capacity in the FSPCMs with coatings (MA/PMMA, 60/40 wt %) confirmed the leakage-proof samples proposed in this study are promising PCMs for practical thermal energy storage applications.

**Figure 6:**
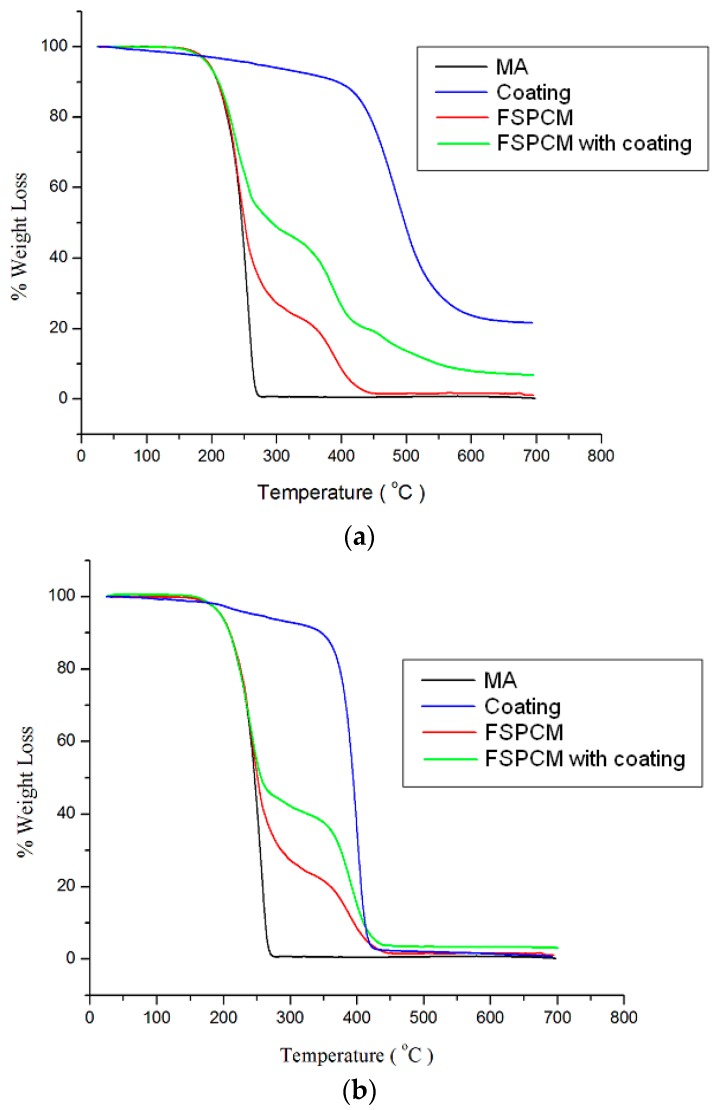
Thermogravimetric analyzer (TGA) curves for (**a**) conformal coating and (**b**) polyacrylic coating.

A thermogravimetric analyzer (TGA) was employed to evaluate the thermal stability of the FSPCM samples. Figure 6 illustrates the TGA curves of pure materials: MA and coatings, and FSPCM with different coatings. Apparently, MA has a single decomposition step that starts at 120 °C and ends at 280 °C. For the FSPCM without coating shown in Figure 6a, there were two steps of thermal decomposition that corresponding to decomposition of MA followed by PMMA. On the other hand, three steps of decomposition can be seen for FSPCM with conformal coating. The first step of decomposition was the decomposition of fatty acid from 120 °C to 300 °C, followed by decomposition of PMMA from 300 °C to 450 °C, and finally the decomposition of conformal coating from 450 °C to 600 °C. Similar decomposition phenomena were noticed for FSPCM with polyacrylic coating in Figure 6b. It is worth noting that the decomposition temperature and process of FSPCM were prolonged with the added coating. This indicates that the working thermal stability of FSPCM can be enhanced by the coating [19]. The TGA curves show both coatings are suitable to be used since their decomposition temperature is higher than that of MA. These findings imply that the coatings can restrain the volatilization of MA and improve the thermal stability of the prepared FSPCMs in the present study.

### 3.4. Morphology Analysis

**Figure 7:**
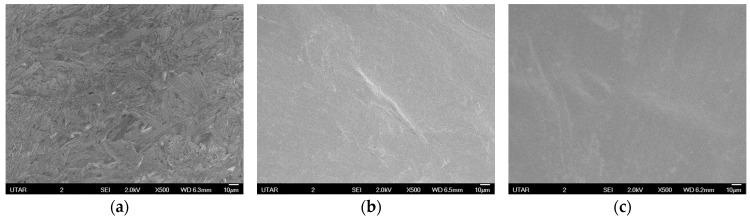
Scanning electron microscope (SEM) photographs of PCM (MA/PMMA, 60/40 wt %): (**a**) without coating; (**b**) with conformal coating and (**c**) with polyacrylic coating.

The surface morphology analysis of the PCMs was conducted by SEM. Figure 7 compares the SEM images at 500 times magnification for various PCMs. Phase separation within the PCMs without coating can be obviously seen in Figure 7a as indicated by the rough surface. However, the surface of PCMs becomes smooth as a thin layer without cracks with the added coatings as shown in Figure 7b,c The observation verified that the PCMs are form-stable against the leakage of fatty acid [20].

### 3.5. Reliability Analysis

**Figure 8:**
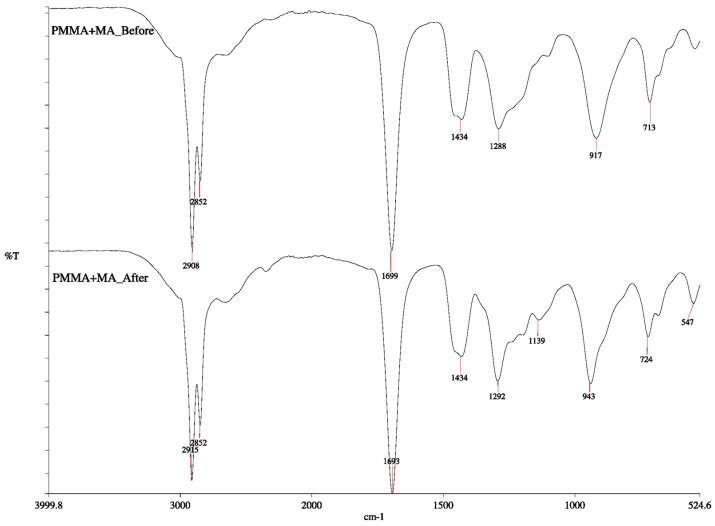
FTIR analysis of the PCM before and after cycling.

PCM must be chemically stable to withstand a number of freezing and melting cycles. In order to evaluate reliability, a 1000 times repeated thermal cycling test was performed using the thermal cycling system. The variance in chemical structure of the PCM after thermal cycling was observed from the FTIR spectra as presented in Figure 8. As can be seen from the spectra of PCM after thermal cycling, almost all peaks are consistent with that of before thermal cycling. This confirms that the chemical structure change is negligible and indicates the good chemical reliability of PCM.

**Figure 9:**
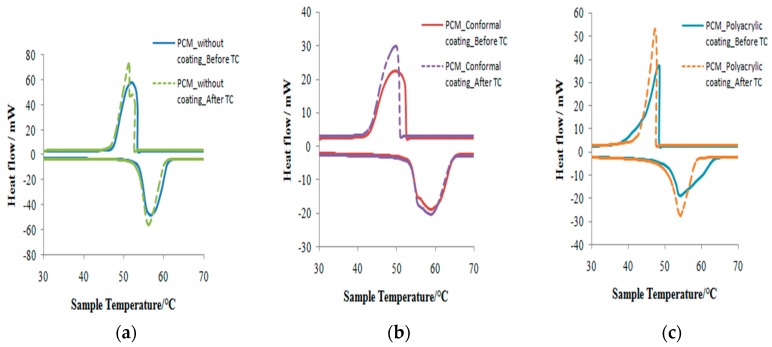
DSC curves of (**a**) PCM without coating; (**b**) PCM with conformal coating and (**c**) PCM with polyacrylic coating before and after 1000 thermal cycling.

For thermal reliability, the properties of PCM after 1000 times repeated thermal cycling were analyzed by DSC. The variances in melting and freezing temperatures before and after thermal cycling were small (−0.68%). In addition, the changes of latent heats of melting and freezing were −2.85% and −3.53%, respectively, indicating the good thermal reliability of the PCM. Similar level of variations could be seen for PCM with coatings. The melting and freezing temperatures changed between −0.02% and −1.40%, whereas the latent heats of melting and freezing dropped within a narrow range of 0.16–1.22% after thermal cycling. The DSC results before and after repeated thermal cycling are given in Table 3 and Figure 9.

## 4. Conclusions 

Composite PCMs with conformal coating and polyacrylic coating were prepared in this study. The leakage test indicated that both coatings can be used to overcome the leakage problem of composite PCMs. The polyacrylic performs slightly better compared to conformal coating. FTIR spectra and SEM images confirmed the PCMs were well coated since only the coating film functional groups were detected after coating and no cracks were observed. For thermal performance, the DSC result showed that the coating did not have much of an effect on melting and freezing points. The TGA curves confirmed that the coatings are able to restrain volatilization of MA and improve the thermal stability of composite PCMs. Furthermore, the coated PCMs exhibited good thermal and chemical reliability after the 1000 repeated thermal cycling. Therefore, the coatings proposed in the present study can potentially be used with PCM as a stable and leakage-free thermal energy storage material in various thermal storage applications for energy conservation.

## Figures and Tables

**Table 1:** Leakage percentages for composite PCMs with and without coatings.

Sample	MA:PMMA	η (%)		
-	(wt %)	Without Coating	Conformal Coating	Polyacrylic Coating
1	20:80	**112.59**	**100.00**	**100.00**
2	40:60	280.67	**100.00**	**100.00**
3	60:40	296.29	**121.02**	**100.00**
4	80:20	616.77	129.40	**113.95**

**Table 2:** Thermal properties of various form-stable PCMs.

Coating	MA:PMMA (wt %)	Melting Point (°C)	Latent Heat of Melting (J/g)	Freezing Point (°C)	Latent Heat of Freezing (J/g)
No coating	20:80	55.30	32.08	52.74	31.85
Conformal coating	20:80	56.15	18.13	49.63	18.44
	40:60	57.09	42.94	51.20	43.13
	60:40	55.34	83.73	52.46	84.07
Polyacrylic coating	20:80	55.41	19.34	48.12	17.41
	40:60	54.93	54.49	51.47	53.68
	60:40	55.02	83.20	50.72	82.08
	80:20	54.19	103.14	48.47	103.27

**Table 3:** DSC results before and after 1000 thermal cycling.

PCM	Melting Point (°C)		Latent Heat of Melting (J/g)		Freezing Point (°C)		Latent Heat of Freezing (J/g)	
	Before	After	Before	After	Before	After	Before	After
Without coating	56.62	56.23	170.65	165.79	51.86	51.51	171.89	165.82
With conformal coating	58.84	58.79	106.12	105.95	49.73	49.64	106.75	106.40
With polyacrylic coating	54.19	54.18	103.14	102.09	48.47	47.79	103.27	102.01
